# Cranial CT scan evaluation of morphological variations and location of pterion in Pakistani male population for lateral neurosurgical approach

**DOI:** 10.12669/pjms.36.3.2003

**Published:** 2020

**Authors:** Aisha Rafi, Sana Sayeed, Muhammad Idrees Anwar

**Affiliations:** 1 Dr. Aisha Rafi, FCPS. Department of Anatomy, Shifa College of Medicine, Shifa Tameer-e-Millat University, Islamabad, Pakistan; 2 Dr. Sana Sayeed, FCPS. Department of Radiology, Shifa College of Medicine, Shifa Tameer-e-Millat University, Islamabad, Pakistan; 3 Dr. Muhammad Idrees Anwar, FRCS. Department of Surgery, Rawalpindi Medical University Rawalpindi, Pakistan

**Keywords:** Cranial CT scan evaluation, Lateral skull approach, Pterion

## Abstract

**Objective:**

To determine the morphological variations and location of pterion in Pakistani male population.

**Methods:**

This retrospective observational study was carried out in the Department of Radiology, Shifa International Hospital from December 2018 to June 2019. The sample size was calculated by Open Epi web-based calculator. Fifty-three cranial CT scans with slice thickness of 0.5mm; consecutive scans of males were randomly selected. The patients with no craniofacial fracture and ages from 25 to 45 years were included. The dataset was obtained from Toshiba Aquilion One, 360-slice MDCT. The images were imported into the imaging software PACS (WFM), and analyzed in maximum intensity projection mode with three dimensional multiplanar reconstruction viewers. Measurements were taken in lateral projections of skull in Frankfurt plane, as horizontal and vertical distance from the posterolateral margin of frontozygomatic suture to center of pterion. Vertical distance from the superior border of zygomaticotemporal arch to the center of pterion. The morphological types were also recognized.

**Results:**

The type of pterion on right side was 94.3% sphenoparietal 5.6% epipteric whereas left side was (90.5%) sphenoparietal (3.7%) epipteric, (3.7%) stellate type, (1.8%) frontotemporal type. The mean horizontal and vertical frontozygomatic measurements on right side were 2.23 ± 0.22cm and 1.25±0.219 cm respectively. The same measurements on the left side were 2.27-±0.25 cm and 1.226-±0.22 cm respectively. The mean zygomaticotemporal measurements on the right and left sides were 3.45 ±0.29cm and 3.44 ±0.25 cm respectively. The mean distance on right and left side of skull was statistically insignificant.

**Conclusion:**

The study provides useful data for position and location of pterion for safe neurosurgical procedures via pterion. Moreover, the knowledge about different morphological types of pterion help the radiologist to differentiate between a fracture line and normal morphological variety.

## INTRODUCTION

Pterion is an H shaped suture lying in the supra temporal fossa, formed by the fusion of frontal, sphenoid, temporal and parietal bones.^1^ This anatomical landmark has been studied since 19th century and the term is derived from the Greek root “pteron” that refers to the wings attached to the head of Hermes, the messenger of the Greek gods.^2^ Pterion is a gate way to important cranial structures for neurosurgical approaches. The peritoneal approach exposes the opercula and Sylvian Fissure, which facilitates the surgeon to access the pathology lying in basal cistern, insula and midline structures like basal ganglia, sellar and parasellar areas, hypothalamic area, third ventricle.^3^ Pterion is an important cranio-metric point used to reach aneurysms and other vascular lesions of the anterior circulation and distal portion of the posterior circulation, as well.^4^ The transcranial surgery for cranio-orbital tumors can be approached through pterion.^5^

Apart from neurosurgery, pterion is important for forensic scientists due to existence of morphological variations in different populations^6^ and forensic anthropologists for age estimation and sex determination.^7^

There is a literature-based evidence that pterion is variable in shape and position. The variation in shape and position is attributed to host of factors, important being, age, sex, side of skull and ethnicity.^8^ Depending on type of bone articulation pattern, Murphy had described four types of pterion,^9^ sphenoparietal, frontotemporal, stellate, and epipteric. A sutural pattern in which the sphenoid and parietal bones directly came together is sphenoparietal variety. When the frontal and temporal bones were in direct contact, a frontotemporal variety. The third one was named as the stellate type, looks like a start at the junction of four bones such as frontal, parietal, temporal and sphenoid bones. The fourth type was named as the epipteric type in which was there was a small sutural bone between the parietal bone and the greater wing of the sphenoid bone. Previously various studies have been conducted to know the exact location of pterion in different ethnic groups e.g.; Turkish male skulls,^10^ Indian skulls^11^ and, Thai dry skulls.^12^

The rationale of the study is that pterion is an important approach for lateral neurosurgical skull procedures and morphological variation might affect the anatomy of the underlying structures and thus the surgical approach. Most of the studies on the morphology and location of pterion are carried out on dry skulls, cranial CT scan investigation of pterion morphology and localization has not been mentioned so far in the literature. The different types of morphological patterns add to the knowledge of radiologist, forensic scientist and anthropologist and provide helpful information to the surgeon for lateral skull approach. The distance of pterion from the nearby reference points has been documented in the dry skulls but measurement by cranial CT scan is not mentioned in the literature.

The objective of this study was to determine the morphological variations and location of pterion in Pakistani male population using cranial CT scan. It also aims to provide useful information to surgeons and radiologists for safe surgical intervention in the area of pterion and differentiate between fracture lines and the normal morphological variation of pterion respectively.

## METHODS

This is hospital-based cross sectional study, carried out in the department of radiology, Shifa International Hospital from December 2018 to June 2019. Sample size was calculated by OpenEpi, a web-based epidemiologic and statistical calculator.^13^

### Inclusion Criteria

In Pakistan traumatic brain injury is common among males varying between 25 to 50 years age.^14^ Therefore, the males between 25 to 45 years of age were included in the study. Pakistani Nationals by birth were selected. Only those males who were presented in the emergency department and the consecutive scans from year 2016 to 2018, with slice thickness of 0.5mm were selected.

### Cranial CT Scans

The institutional review board STMU (Shifa Tameer-e-Milat University) permission was sought before the start of study (IRB#1078-354-2018, August 8, 2018). The 53 cranial CT scans were randomly selected, with slice thickness of 0.5mm, consecutive scans of males from year 2016 to 2018, performed in Shifa International Hospital, department of diagnostic radiology. These all males underwent CT scan when referred from ER, undergoing workup for road traffic accident injury or fall and 0.5mm slice thickness of bone window is included in routine cranial CT per departmental protocol, only those patients were selected who have no craniofacial fracture, with ages from 25 years to 45 years, confirmed by signed reports as well. Radiologist assesses an optimal image of pterion, and if pterion is not optimally visualized then the next random case is selected with all above inclusion criteria.

The dataset was obtained from CT scanner Toshiba Aquilion One, 360-slice MDCT using 0.5mm slice thickness. The images were imported into the imaging software PACS (WFM), and analyzed in bone window and maximum intensity projection mode with three dimensional multi planar reconstruction views using 90mm slab thickness to obtain best surface rendered images. Lateral projections of skull were used for imaging of pterion in Frankfurt plane using the reference points with optimal visualization of frontozygomatic suture, zygoma and pterion. About 10% of the cases measurements are repeated randomly.

**Fig.1:**
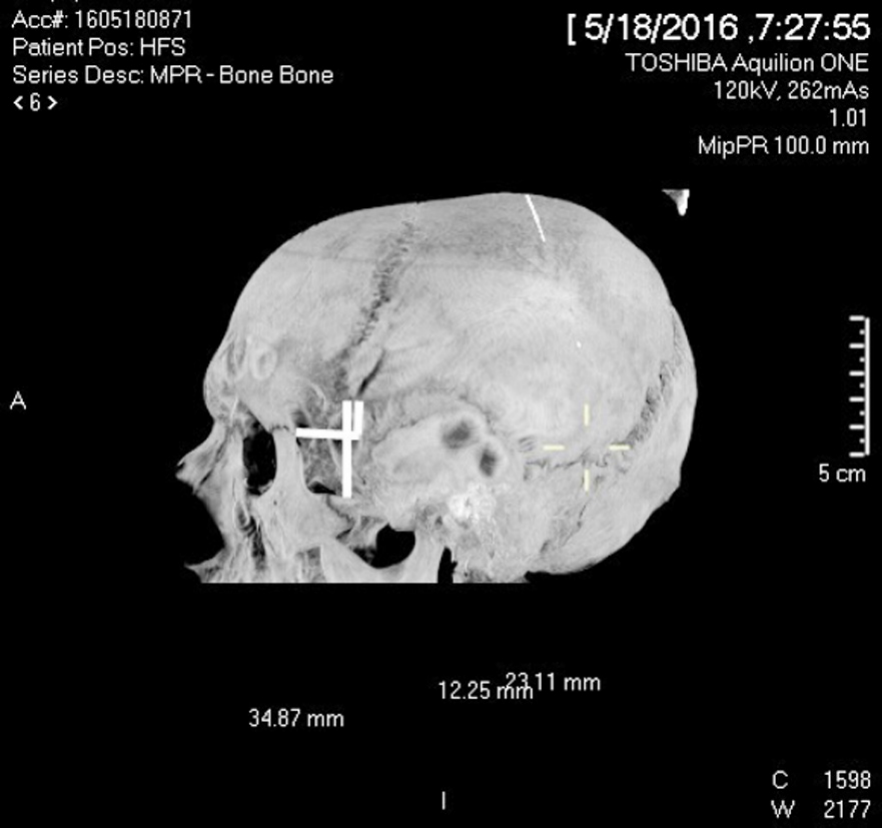
Measurements taken from the midpoint of pterion. 1: Horizontal distance from posterolateral aspect of frontozygomatic suture. 2: Vertical distance from posterolateral aspect of frontozygomatic suture. 3: Vertical distance from zygomaticotemporal arch.

Following variables or measurements were taken both on the right and left sides in cranial CT scan^15^ (Fig 1).

1. ***Frontozygomatic (Horizontal):*** Horizontal distance from the posterolateral margin of frontozygomatic suture to center of pterion.
2. ***Frontozygomatic (Vertical):*** Vertical distance from the posterolateral margin of frontozygomatic suture to center of pterion
3. ***Zygomaticotemporal (Vertical):*** Vertical distance from the superior border of zygomatic arch to the center of pterion.

## RESULTS

### Morphological types of pterion

**Fig.2:**
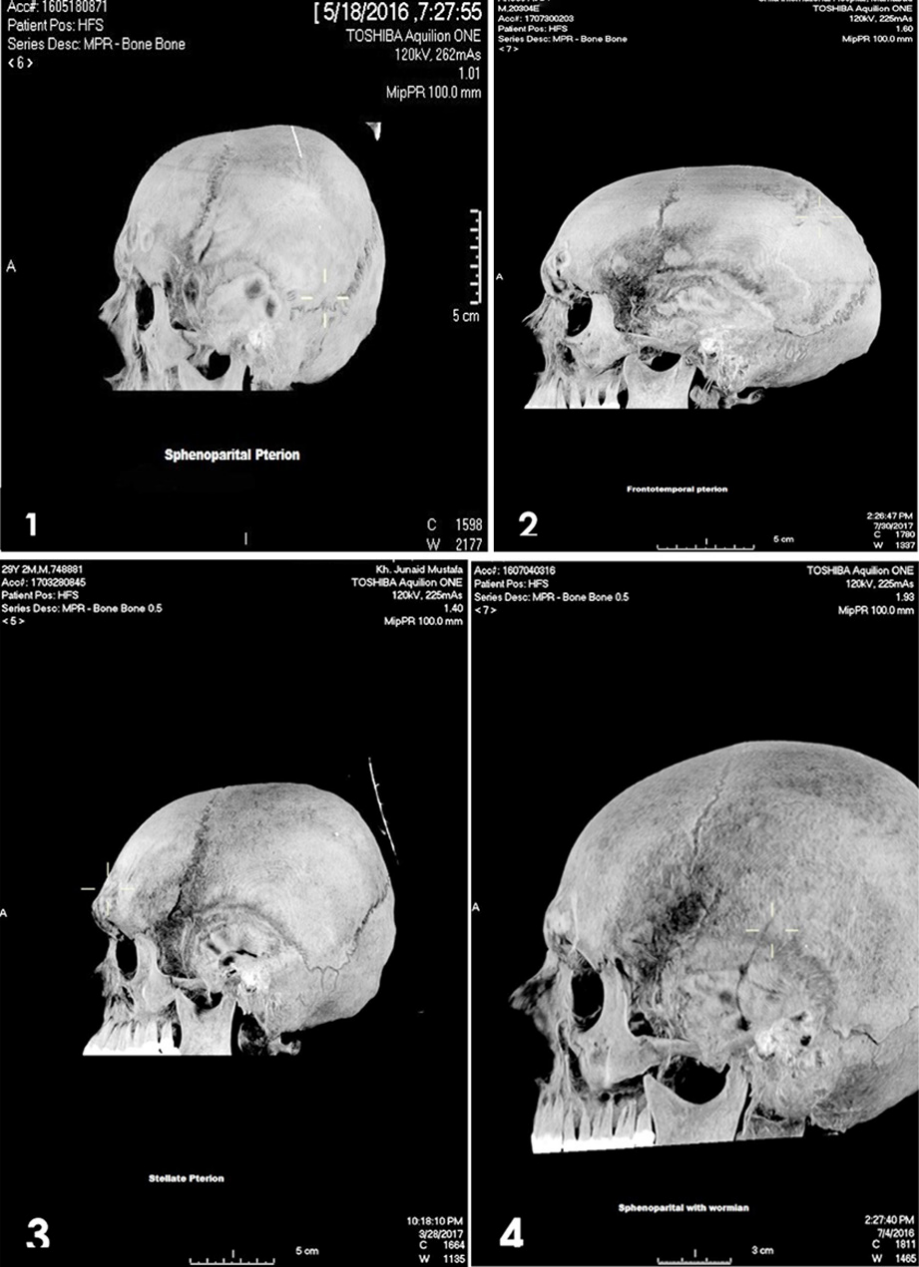
Morphological variations of pterion. 1: Sphenoparietal. 2: Frontotemporal. 3: Stellate. 4: Epipteric.

The types of pterion were assessed as percentages and frequency (n). In our sample of cranial CT Scan of 53 males, we assess 106 pterions (right and left side). The type of pterion on right and left side was different. On the right side 94.3%(n=50) were sphenoparietal type where all the bones join at an H shaped suture. 5.6%(n=3) were epipteric type, where an intersutural bone is found in the pterion (Fig.2) (Table-I).On the left side of the skull the cranial CT scan reveal 48(90.5%) of sphenoparietal variety, 2(3.7%) were epipteric and 2(3.7%) were stellate type, 1(1.8%) was frontotemporal type (Fig.2) (Table-I).

**Table-I:** Percentage and number of different types of pterion.

Morphological types	Right side		Left side	

	Number	Percentage	Number	Percentage
Sphenoparietal	50	94.3%	48	90.4%
Epipteric	3	5.6%	2	3.7%
Stellate	-		2	3.7%
Frontotemporal	-		1	1.8%

### Location of pterion

The data was entered in SPSS version 23. The continuous variables were horizontal and vertical distance of pterion from posterolateral margin of frontozygomatic suture. The vertical distance from the zygomaticotemporal arch to the midpoint of pterion was also measured. The pair sample t test was applied to compare the mean distance of pterion from the above reference points on the right and left side of skull.

The mean horizontal and vertical frontozygomatic measurements on the right side were 2.23 ± 0.22cm and 1.25±0.219cm respectively. The mean horizontal and vertical frontozygomatic measurements on the left side were 2.27±0.25 cm and 1.226 ±0.22 cm respectively (Table-II). The Mean ± SD measurements on both sides were statistically insignificant. It means that pterion lies 2.23 cm posterior and 0.22 cm above the frontozygomatic suture on the right side of the skull in Pakistani males. The pterion on the left side of skull lies 2.27±0.25 cm posterior and 1.226 ±0.22 cm above the frontozygomatic suture in Pakistani males.The mean zygomaticotemporal measurements on the right and left sides were3.45 ±0.29cm and 3.44 ±0.25 cm respectively and were not significantly different with a p-value of 0.79. (Table-II)

**Table-II:** Mean distance of Pterion from the fromtozygomatic suture and zygomaticotemporal arch.

Measurements	Mean (cm)	Standard Deviation	Paired Samples t Test		

			95% Confidence Interval of the Difference		p-Value

			Lower	Upper	
Right Frontozygomatic (Horizontal)	2.230	0.227	-0.109	0.018	0.162
Left Frontozygomatic (Horizontal)	2.275	0.253			
Right Frontozygomatic (Vertical)	1.253	0.219	-0.028	0.081	0.336
Left Frontozygomatic (Vertical)	1.226	0.225			
Right Zygomaticotemporal (Vertical)	3.458	0.298	-0.062	0.081	0.795
Left Zygomaticotemporal (Vertical)	3.449	0.257			

## DISCUSSION

The cranial CT scans for evaluation of pterion, a craniometrical point of considerable significance, map the pterion for minimal invasive surgical approach for a number of procedures.^4^,^5^ This study defined the radio logically detectable variation of pterion in Pakistani population. Males were selected, because most of the victims of road traffic accidents in Pakistan are males. Traumatic brain injury management needs improved neuroimaging facilities.^16^ The radiologists should know the variations of pterion to differentiate between fracture line and suture to make an accurate diagnosis and neurosurgeons for performing surgery at pterion.^17^ The Cranial sutures undergo a series of morphological changes from birth till childhood owing to growth of brain.^18^ Although the true determinants of the sutural pattern formed by the articulation of cranial bones are not fully known. The cranial bone morphogenesis is under influence of homeobox gene, MSX2.It might cause ethnic and racial variations of suture patterns.^19^ The morphology of pterion is important for anatomists, anthropologists, and forensicpathologists.^20^ The preoperative planning for brain tumor excision requires an immaculate knowledge of anatomy of brain and skull. The preoperative planning for resection of brain tumors is largely dependent on knowledge about palpable bony landmarks and careful measurements by radiological scans as an objective guide to resection. Thus, Pterion is an important craniometric point and its variant morphology and location has an important impact on pre-operative neurosurgical planning.^21^

Morphometric analysis using modern radiological techniques such as CT scan gives an accurate measurement. This study showed that distance of pterion from the reliable reference points of skull is almost same on both sides. The Mean ± SD measurements on both sides were statistically insignificant. This shows the symmetrical position of pterion in both halves of skull, no matter the type of pterion. The study also concludes that pterion is formed at more or less the same location during embryogenesis and the type of pterion does not affect it. The studies carried out on dry skulls have same results that there is no significant difference in the distance of pterion from frontozygomatic suture and zygomaticotemporal arch on right and left skull of different ethnic groups.^10^,^11^ The results of our study correspond very well with the studies conducted on the dry human skulls in different time, race and country where research took place.

The predominant type of pterion in this study is sphenoparietal variety (90.5%), which is not only present in majority of male population in Pakistan but it is also a predominant variety in most of the ethnic groups, reported by Zawaldia et al.^22^ who analyzed the type of pterion in eight different ethnic groups. The sphenoparietal variety is found in different population groups ranging from 66% to 95.3%.^22^ The results correspond with the results of our study.

This study showed that the epipteric variety is found in 5.6% of Pakistani male skulls. Epipteric variety is formed duet to the presence of wormain or intersutural bones at the pterion.^23^ There is a negligible significance of morphological variety but some researchers associate the epipteric bone formation with the genetic factors. Osteogenesis imperfecta and cleidocranial dysostosis can be accompanied by wormian bones.^24^ The sutural bones become significant while interpretation of CT scan and radiographs of patients presented with traumatic head injury. The radiologists have to make the differential diagnosis between sutural bones and fractures. The morphological variations of cranial suture might lead to confusion while reporting the bone windows in CT scans.^25^ The presence of epipteric bone at pterion might lead to difficulty in making surgical burr hole in the region. This necessitates that neurosurgeons and radiologists should know the presence and incidence of these structures in general and Pakistani population in particular.

Another variation of pterion found in this this study is stellate type (2.7%), found in the cranial CT scans of only two males. There is a higher incidence of stellate shape pterion in Nigerians (5.0%)^26^ and West Anatolians (5.5%).^27^

The frontotemporal variation of pterion is present on one side of skull in only one skull (1.8%). in this study. There is less incidence of frontotemporal pterion in Pakistani population compared to Turkish (10%)^10^ and South Indian (3.5%)^11^ populations. Frontotemporal variant of pterion is found in primates’ skull,^28^ having small brains. The humans have larger brain so predominantly they have sphenoparietal variety.

In our study stellate type and frontotemporal pterion is found in two and one skulls respectively on left side, the other side has sphenoparietal type of pterion in all the three cases. The degree of association between type of pterion type and sideness was not statistically significant in one of the study carried on Nigerian skull.^25^ It can be explained by the fact that morphological pattern of suture is under control of MSX gene^17^ that might be expressed differently on both sides of skull.

The knowledge of morphological variations of pterion is important because in epipteric variety the burr hole placed over the anterior junction for draining extradural hematoma might penetrate the orbit.^29^ The forensic scientists and anthropologists used pterion as an anterolateral landmark to determine age and sex of corposes.^30^ This study identifies morphological variations of pterion, which will help to avoid the potential morbidity; and mortality related to false-negative interpretation of fractures as normal sutures.

### Limitation of the study

It needs to be explored that morphological variation of pterion is reflected in the underlying anatomical position of the cranial structures. It’s a retrospective observational study on Pakistani male population only.

## CONCLUSION

Morphometric analysis of pterion using modern radiological techniques such as the cranial CT revealed morphological variations of pterion present in Pakistani population. The results of this study will be useful for neurosurgeons for planning safe neurosurgical approach, for radiologists in differentiating the fracture from normal morphology and for forensic experts in assessing the type and location of the pterion in incomplete archeological remains or forensic materials.

### Author`s Contribution:

**AR** conceived, designed and did statistical analysis & editing of manuscript.

**SS** did data collection and manuscript writing.

**MIA** did review and final approval of manuscript.

All the three authors, are responsible for integrity and authenticity of work.
